# The 2024 National Academies of Sciences, Engineering, and Medicine Long COVID Definition: What Clinicians Need to Know

**DOI:** 10.1007/s11606-025-09415-8

**Published:** 2025-03-10

**Authors:** Lily Chu, Karyn Bishof, Abigail A. Dumes, E. Wesley Ely, Paule V. Joseph, Andrea B. Troxel, on behalf of the National Academies of Sciences, Engineering, Medicine (NASEM) Committee on Examining the Working Definition for Long COVID

**Affiliations:** 1 International Association for Chronic Fatigue Syndrome/Myalgic Encephalomyelitis, Stony Brook, NY USA; 2 COVID-19 Longhauler Advocacy Project, Inc. (C19LAP), Boca Raton, FL USA; 3 Department of Women’s and Gender Studies, University of Michigan, Ann Arbor, MI USA; 4 Critical Illness, Brain Dysfunction, and Survivorship (CIBS) Center, Vanderbilt University Medical Center, Nashville, TN USA; 5 Geriatric Research Education Clinical Center (GRECC), Veteran’s Affairs Tennessee Valley Healthcare, Nashville, TN USA; 6 National Institute on Alcohol Abuse and Alcoholism, National Institute on Deafness and Other Communication Disorders, National Institutes of Health, Bethesda, MD USA; 7 Division of Biostatistics, Department of Population Health, NYU Grossman School of Medicine, New York, NY USA

**Keywords:** diagnosis, Long COVID, definition, post-acute sequelae of COVID-19, COVID-19

## Abstract

Millions of Americans affected by Long COVID (LC) report difficulty accessing care and support. One barrier is obtaining a diagnosis. In response, US federal agencies commissioned a National Academies of Sciences, Engineering, and Medicine (NASEM) committee to re-examine the existing federal definitions for LC. The Committee concluded that LC is “an infection-associated chronic condition (IACC) occurring after SARS-CoV-2 infection that is present for at least 3 months as a continuous, relapsing and remitting, or progressive disease state that can present as singular or multiple symptoms and/or diagnosable conditions.” The full report was released in June 2024. We briefly highlight features and aspects of the definition that may help clinicians identify those who remain undiagnosed and improve care for all LC patients.

For 5% of American adults, Long COVID (LC) is a persistent issue, with 80% of this population reporting functional limitations.^1^ Despite this high disease burden, people with LC face challenges in accessing adequate care and support. In response, US federal agencies commissioned the National Academies of Sciences, Engineering, and Medicine (NASEM) to convene a committee to re-examine, harmonize, and update the existing federal definitions for LC.

Based on a review of scientific literature, expert testimony, and patients’ lived experiences, the Committee defined LC as “an infection-associated chronic condition (IACC) occurring after SARS-CoV-2 infection that is present for at least 3 months as a continuous, relapsing and remitting, or progressive disease state.”^2^ It affects one or more organ systems, presenting as single or multiple symptoms and/or diagnosable conditions.

The Committee’s full report was released in June 2024 and is available on the NASEM website.^2^ Figure 1 summarizes the definition and its key features. For more information about the process, evidence, and reasoning behind the definition, please refer to the report or to the July 2024 publication in the *New England Journal of Medicine*.^3^ A section of the report also explains how to use the definition in clinical care, research, and public health surveillance.

**Figure 1:**
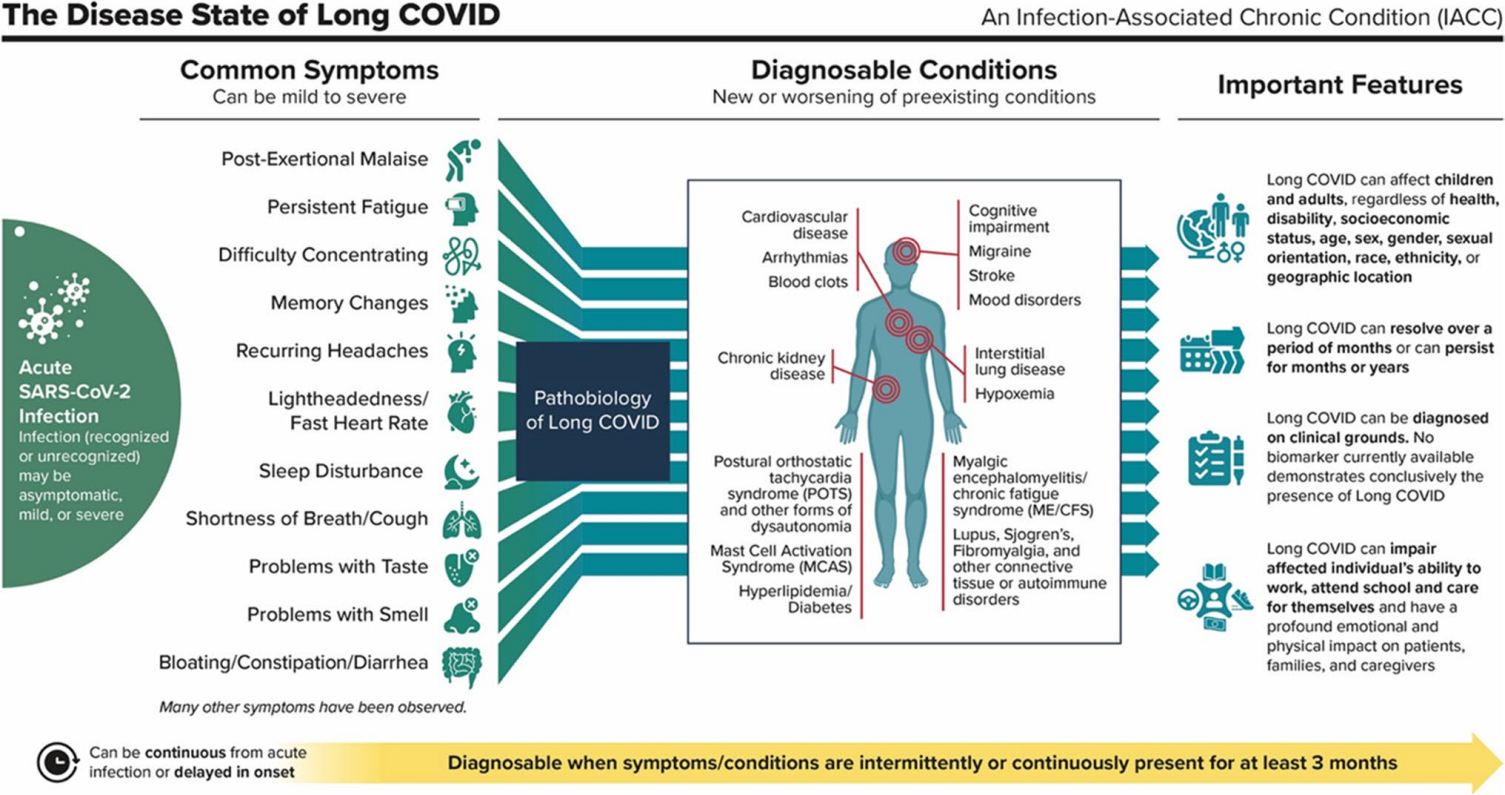
The disease state of Long COVID, an infection-associated chronic condition.

Previously healthy individuals who have reported acute symptoms consistent with SARS-CoV-2 infection, have documentation of an abnormal SARS-CoV-2 test, and endure continuous symptoms readily trigger suspicion of LC. However, many people affected by LC do not fit this profile. For example, some describe a “honeymoon” phase of recovery following acute infection only to have chronic symptoms emerge later. Others present with unexpected deterioration of pre-existing conditions or, months later, with de novo conditions like postural orthostatic tachycardia syndrome (POTS) or scleroderma. These are all possible manifestations of LC. In this article, we highlight how clinicians can use the 2024 NASEM Long COVID Definition to diagnose those who remain undiagnosed and to improve care for all LC patients.

1. Anyone can be affected by LC.

LC can affect children and adults, regardless of health, disability, socioeconomic status, age, gender, sex, ethnicity, race, sexual orientation, or geographic location. Physicians should be aware of social biases and stereotypical assumptions that might prevent them from diagnosing patients and work to ensure that marginalized and underserved individuals are not overlooked.

2. Although LC patients may suffer from diverse symptoms related to many organ systems, some symptoms are more common than others. No specific symptom is required for a diagnosis of LC, but the presence of common symptoms increases the likelihood of LC.

LC patients may experience one or more of the following symptoms (Fig. 1): fatigue, post-exertional malaise (PEM), difficulty concentrating, memory changes, recurring headache, shortness of breath, cough, persistent lightheadedness, fast heart rate, sleep disturbance, problems with taste or smell, bloating, constipation, and diarrhea.^2^

PEM is immediate or delayed exacerbation of some or all of a patient’s LC symptoms following cognitive, physical, emotional, orthostatic, or sensory challenges.^4^ The intensity and duration of the exacerbation are often out of proportion to the trigger. For example, walking a block or reading instructions can lead to prolonged worsening of a person’s memory and executive function.

This list of symptoms is neither mandatory nor exhaustive; LC patients exhibit many other symptoms.^5^ However, new onset or, in the case of individuals with pre-existing medical conditions, exacerbation of these symptoms can reveal an LC diagnosis.

3. Symptoms must be present for at least 3 months. This period can occur at any time after infection. Symptoms may improve, worsen, stabilize, or disappear and reappear over this period.

Studies and public input suggest that people who have not recovered by 3 months are likely to still be sick at 1 year.^6^ A 3-month period allows for the resolution of temporary symptoms, assessment for alternative causes of symptoms, and therapeutic trials. Because the LC disease state can begin weeks to months after apparent full recovery, the 3 months need not begin during or immediately after the acute SARS-CoV-2 infection. In one longitudinal study, a third of participants reporting complete remission experienced recurrence and/or appearance of new symptoms over time.^6^ Furthermore, Davis et al. noted that among study participants reporting cognitive issues, 43% experienced them a month or more after their acute SARS-CoV-2 infection.^7^ Parosmia did not begin until 3 months after infection and musculoskeletal pain, paresthesia, and hair loss were more common at 1 year than at 2 months. Time should be counted from when symptoms begin. Symptoms may not be continuous but may instead remit and relapse. These features of the definition enable diagnosis of patients who did not develop symptoms immediately or continuously after an acute infection.

4. LC can follow mild or even asymptomatic infections. A positive COVID test is not required to make an LC diagnosis.

People with LC may lack proof of a positive COVID test due to variable test availability (especially at the beginning of the pandemic), barriers to testing, initially asymptomatic infections, limited test sensitivity, a decline in rates of reporting, and/or waning immunity.^2^ Exposure to someone with a positive test and memory of an infection-like episode accompanied by symptoms consistent with acute SARS-CoV-2 infection increase the probability of prior SARS-CoV-2 infection. Symptom type, quality of life, disease trajectory, and results of cognitive testing do not appear to differ between those with and without a positive test.^5,8^ In one study, 41% of study participants without a positive commercial COVID test showed evidence of infection on research-based assays.^8^

5. To date, no test or biomarker definitively confirms LC. Diagnosis can be made based on history, physical examination, and limited diagnostic testing.

All symptoms should be clinically evaluated by the treating physician to look for co-existing medical conditions and alternative diagnoses. Testing should not be ordered in an indiscriminate manner but selected based on an individual patient’s presentation and with potential alternative diagnoses in mind. A thorough review by the Committee found that, to date, there are no validated tests to diagnose LC. Recently, Erlandson et al. examined twenty-five different routine laboratory tests and found no difference between those who had and had not recovered from their SARS-CoV-2 infection.^9^

6. Co-existing diagnosable conditions are common. Their presence does not exclude a diagnosis of LC.

LC is not a diagnosis of exclusion and can exist alongside pre-existing and newly developed medical conditions. Ultimately, each clinician must determine the best workup for a patient’s complaints and when it is appropriate to attribute a patient’s constellation of symptoms to LC.

The definition lists several medical conditions (Fig. 1) that have emerged among LC patients at high rates. For example, a meta-analysis found that 51% of LC patients fit myalgic encephalomyelitis/chronic fatigue syndrome (ME/CFS) criteria and one study found that 79% may be affected by postural orthostatic tachycardia syndrome (POTS), a manifestation of autonomic dysfunction.^10,11^ Using large medical databases, researchers in Germany, Taiwan, and the UK have each found an increased risk of autoimmune disease among those previously infected by SARS-CoV-2.^3^

Patients have reported that some clinicians would no longer consider LC after a diagnosis with another condition, even if that condition did not account for all of their symptoms and/or followed on the heels of SARS-CoV-2 infection. Furthermore, identifying and treating these disorders can improve patients’ lives, even in the absence of a disease-modifying treatment for LC. Management techniques, such as activity pacing for PEM and compression garments for POTS, can be applied immediately as they are low-cost and pose minimal risk.

Conditions such as ME/CFS, POTS, mast cell activation syndrome, and autoimmune disorders (e.g., rheumatoid arthritis, diabetes mellitus) should be considered as part of the patient’s LC disease state. All diagnoses should remain in the patient’s medical record. If biomarkers or therapies for LC become available, they should be considered for these patients to better characterize or improve their health.

7. LC often affects function negatively. The type and degree of impairment can vary.

Twenty-five percent of LC patients report significant impairment in their daily lives.^1^ Assessment and management of impact through referrals to occupational therapy may improve function. Obtaining disability benefits can be particularly difficult for LC patients.^12^ Documenting the link between symptoms and function can support applications for work/school accommodations and disability benefits. Physicians can also refer to the Social Security Administration’s guidance document for LC.^13^

8. While some LC patients recover within months, others may remain sick for years.

LC patients’ symptoms are often dismissed, downplayed, or attributed to psychological causes because of symptom non-specificity, lack of biomarkers, and the assumption that infected but non-hospitalized patients recover fully and quickly. A significant number of patients and people with LC symptoms have been ill since 2020.^14^

It was challenging to create this definition because there are many areas where the evidence is non-existent, sparse, or evolving. Nonetheless, the Committee was able to extract features of LC that are important for clinicians to recognize now. People with diseases that affect multiple organ systems, are insufficiently understood, and/or for which no simple diagnostic tests are available are frequently left without a diagnosis for months to years.^15,16^ These patients should also be monitored regularly for changes in health that may signal an alternative or additional diagnosis.

The Committee recommended that the federal government adopt, implement, publicize, and refine the definition. Accordingly, the US Centers for Disease Control and Prevention have already begun using the definition and the federal Long COVID Coordination Council has approved it for general use.^17,18^ The Council will analyze and address application of the definition in clinical settings.

The Committee also recommended revisiting the definition in no more than 3 years or whenever significant, relevant findings emerge. A section of the report delineates shortcomings in the evidence base and issues that need clarification. In the meantime, prompt diagnosis of LC is critical so that those affected may receive appropriate care, education, validation, and assistance.
